# A model of influences on the clinical learning environment: the case for change at one U.S. medical school

**DOI:** 10.1186/s12909-017-0900-9

**Published:** 2017-03-23

**Authors:** Howard B. Fleit, Richard J. Iuli, Janet E. Fischel, Wei-Hsin Lu, Latha Chandran

**Affiliations:** 1 Department of Pathology, Stony Brook University School of Medicine, Stony Brook, NY 11794-8691 USA; 2 Stony Brook University School of Medicine, Stony Brook, USA; 3 Department of Pediatrics, Division Chief, Developmental and Behavioral Pediatrics, Stony Brook Children’s Hospital, Stony Brook University School of Medicine, Stony Brook, USA; 4 Department of Family, Preventive and Population Health, Stony Brook University School of Medicine, Stony Brook, USA; 5 Donoho Academy of Clinical and Educational Scholars, Stony Brook University School of Medicine, Stony Brook, NY USA

**Keywords:** Clinical learning environment, Medical student mistreatment, Professionalism, Institutional leadership, Patient safety

## Abstract

**Background:**

The learning environment within a school of medicine influences medical students’ values and their professional development. Despite national requirements to monitor the learning environment, mistreatment of medical students persists.

**Methods:**

We designed a program called WE SMILE: *We can Eradicate Student Mistreatment In the Learning Environment* with a vision to enhance trainee and faculty awareness and ultimately eliminate medical student mistreatment. We provide a description of our program and early outcomes.

**Results:**

The program has enhanced student awareness of what constitutes mistreatment and how to report it. Faculty members are also aware of the formal processes and procedures for review of such incidents. Our proposed model of influences on the learning environment and the clinical workforce informs the quality of trainee education and safety of patient care. Institutional leadership and culture play a prominent role in this model. Our integrated institutional response to learning environment concerns is offered as a strategy to improve policy awareness, reporting and management of student mistreatment concerns.

**Conclusions:**

Our WE SMILE program was developed to enhance education and awareness of what constitutes mistreatment and to provide multiple pathways for student reporting, with clear responsibilities for review, adjudication and enforcement. The program is demonstrating several signs of early success and is offered as a strategy for other schools to adopt or adapt. We have recognized a delicate balance between preserving student anonymity and informing them of specific actions taken. Providing students and other stakeholders with clear evidence of institutional response and accountability remains a key challenge. Multiple methods of reporting have been advantageous in eliciting information on learning environment infringements. These routes and types of reporting have enhanced our understanding of student perceptions and the specific contexts in which mistreatment occurs, allowing for targeted interventions. A common platform across the healthcare professions to report and review concerns has afforded us opportunities to deal with interprofessional issues in a respectful and trustworthy manner. We offer a model of learning environment influences with leadership and institutional culture at the helm, as a way to frame a comprehensive perspective on this challenging and complex concern.

**Electronic supplementary material:**

The online version of this article (doi:10.1186/s12909-017-0900-9) contains supplementary material, which is available to authorized users.

## Background

Historically, reports of inappropriate learning environments, abuse and mistreatment of medical trainees abound. While the earliest report of abuse of medical interns dates to 1928 [1], two commentaries published over thirty years ago brought this issue to heightened attention [2, 3] resulting in several studies of medical student perceptions of mistreatment at individual medical schools [4–7]. In 1992, the Association of American Medical Colleges (AAMC) began to include questions related to this problem in the graduation questionnaire (GQ) that is offered to all U.S. graduating medical students for completion [8]. Later, the AAMC defined specific behaviors in the faculty trainee relationship in their teacher-learner compact [9]. The Liaison Committee on Medical Education (LCME), the accrediting body of U.S. medical education programs, and the Committee on the Accreditation of Canadian Medical Schools (CACMS) have recommended that medical schools “define the standards of conduct in the teacher-learner relationship, develop procedures to address complaints that are received confidentially and devoid of retaliation, and develop educational programs to prevent the behaviors.” Currently, two LCME standards (Standards 3.5 and 3.6) address the learning environment and student mistreatment issues [10]. The predominant behaviors that have emerged from the AAMC GQ over this time period have been incidents of public humiliation and belittlement, and sexual and racial harassment. The individuals primarily responsible for these behaviors have consistently been reported as attending physicians, residents, and nurses [4, 7]. As such, priority ought to be given to the clinical learning environment in our effort to impact trainee experiences and professionalism favorably [11].

Leape et al. suggest that patient safety is adversely affected by a dysfunctional culture derived from disruptive behaviors, including humiliating treatment of students, residents and nurses, passive-aggressive behavior, dismissive treatment of patients, and overall disrespect [12]. Patient safety and quality of care are dependent on several professional characteristics that should be highly valued, such as clear exchange of information, seamless teamwork, a climate that encourages questioning, and monitoring the often complex and nuanced steps toward preferred patient outcomes. Those serving in supervisory roles in the hierarchies of medicine can certainly provide strong leadership and exemplary role modeling, but unfortunately, some might also promote and promulgate disrespect and dismissive, unprofessional or aggressive behavior [12, 13]. Some have argued that perhaps it takes only a few, but saliently inappropriate, authority figures to have influence on learners. However, trainee focus group distillations, surveys, and broader examination of the behaviors of health sciences faculty suggest that unprofessional or uncivil behavior interpretable as mistreatment is a common and frequent experience [14–17]. Others have provided perspective on the pressures, tensions, and even temperaments of those entering medicine as risk factors for unprofessional behavior [12, 18, 19]. Still others have warned that we need to attend to “unintended learning” as well as what is intended [20].

There are likely several contexts in which we might frame the persistence of mistreatment in the learning environment. Social learning theory offers strong evidence on the potency of the behavior of role models, and on the precept that aggression likely begets aggression [21]. Additionally, the transactional model of development might provide a framework for ways in which salient role models impact an individual’s or indeed a team’s behavior, changing it somewhat, and, in turn, influencing the role model (s) and the team’s behaviors in an iterative onward process. [22]. Such bidirectional influences are complicated in medicine by differences in power or stature, the relatively high stakes environment of clinical care, and the emergence of strong influences to maintain a culture that might claim to value transparency or accountability but may not embrace these values with regard to professional and respectful behavior. Indeed, a recent commentary in *Academic Medicine* provides a resident perspective on the saliency of influences that work to perpetuate mistreatment and the acculturation of uncivil behavior [23]. Another perspective from which to view these relationships is analogous to a biochemical cascade, in which an upstream activity is amplified on the subsequent agents. Thus, the inappropriate behavior of the attending could likely get amplified as it impacts the residents and other clinical staff and then to the students, ultimately creating risks for safe patient care.

Despite the attention by accrediting bodies, as well as genuine efforts at individual schools, mistreatment of medical students in the learning environment has remained pervasive. A report based on a thirteen-year study at one medical school revealed that in spite of efforts to educate faculty and residents, create policies to prevent mistreatment, and develop mechanisms to report mistreatment, the problem has persisted [24]. A summary of twelve years of data derived from the AAMC GQ [25], a subsequent systematic review and meta-analysis [26], and a more recent survey of 28 U.S. medical schools [27], confirm that medical student mistreatment remains a problem within medical schools worldwide. Longstanding efforts to improve the learning environment have largely been in vain; medical students continue to report experiencing verbal and physical abuse, as well as sexual and racial discrimination globally [28]. In a recent guide to optimizing graduate medical education, the AAMC has identified the need to nurture optimal learning environments at medical schools as a priority [29].

In 2009 during Stony Brook University School of Medicine’s preparation for its 2011 LCME accreditation review, two key deficiencies were recognized: 1) student and faculty lack of awareness of what constituted mistreatment in the learning environment; and 2) the absence of a systematic approach to report, review and adjudicate student reports of mistreatment. In response, we actively sought to create a multifaceted approach to combat the problem. A team of medical education leaders set about to create the *WE SMILE* program – We can Eradicate Student Mistreatment In the Learning Environment. Our goals were to enhance awareness of what constitutes mistreatment and what does not, and to develop and expand mechanisms for safe reporting, analysis, and resolution of mistreatment issues. In this article, we describe the *WE SMILE* program, which includes education, reporting, reviewing and adjudication, enforcement, and communication. We share this information and the lessons learned along the way so that other institutions might adopt or adapt it to address their learning environment and student mistreatment challenges.

## Methods

### The *WE SMILE* program

We created six distinct steps in the *WE SMILE* program. They include: 1) definition of mistreatment and clear statement of behavioral expectations of administration, faculty and trainees; 2) education of all participants in the learning environment; 3) establishment of anonymous and confidential ways to report mistreatment or learning environment concerns; 4) mechanisms to review and fairly adjudicate reported incidents; 5) monitoring and enforcement; and 6) closing of the loop by communication of outcomes of reporting.

### Definition of mistreatment and expectations of appropriate behavior

An important and fundamental starting point is a shared understanding of the definition of mistreatment and the associated explicit expectations of teachers and learners. The 2011 AAMC GQ included a definition of mistreatment as a preamble to the 13 questions regarding medical student mistreatment: “Mistreatment, either intentional or unintentional, occurs when behavior shows disrespect for the dignity of others and unreasonably interferes with the learning process: Examples of mistreatment include sexual harassment; discrimination or harassment based on race, religion, ethnicity, gender, or sexual orientation; humiliation, psychological or physical punishment and the use of grading and other forms of assessment in a punitive manner.” *WE SMILE* defines mistreatment as “physical, verbal or emotional behavior that shows disrespect for medical students and unreasonably interferes with their learning process.”

In order to maintain an environment that promotes academic and professional success in learners and teachers at all levels, we developed a teacher-learner compact adapted from the AAMC compact between resident physicians and their teachers and the Vanderbilt University School of Medicine’s compact between teachers and learners [9, 30]. Achievement of such success is dependent upon an environment free from behaviors that can undermine important elements of the institution’s mission. Teachers and learners bear significant responsibility for co-creating and maintaining this atmosphere. Teachers of medicine also bear particular responsibility with respect to their evaluative roles relative to student work and to their modeling of appropriate professional behaviors. Our students sign this compact (available from the authors upon request) before receiving their white coats during the first week of medical school. All faculty members involved in teaching sign the same compact electronically in the beginning of each academic year.

### Education of stakeholders

We developed a set of teaching scenarios that depict examples of behaviors in the clinical environment that may or may not represent student mistreatment. We use these scenarios to trigger interactive small group discussions. We created a PowerPoint presentation with embedded learning environment scenarios for training purposes. Education in the form of interactive seminars and workshops is provided in various forums including departmental faculty meetings, student orientation and class meetings, nursing leadership meetings and special joint meetings with residents and faculty. The presentation is also made available online for asynchronous access.

### Mechanisms for safe reporting of concerns

**Fig. 1:**
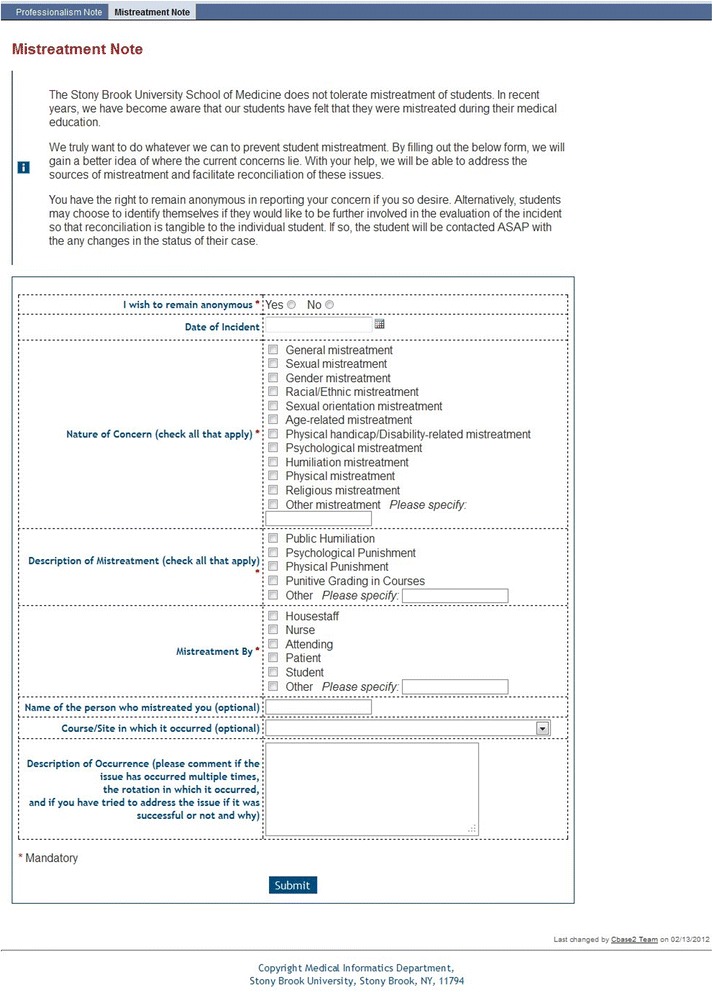
Mistreatment Incident Reporting Form. Students may access this form from the school’s online information management system. Following submission, the form is sent via email to the Associate Dean for Student Affairs and the Vice Dean for Academic and Faculty Affairs for review and triage

**Fig. 2:**
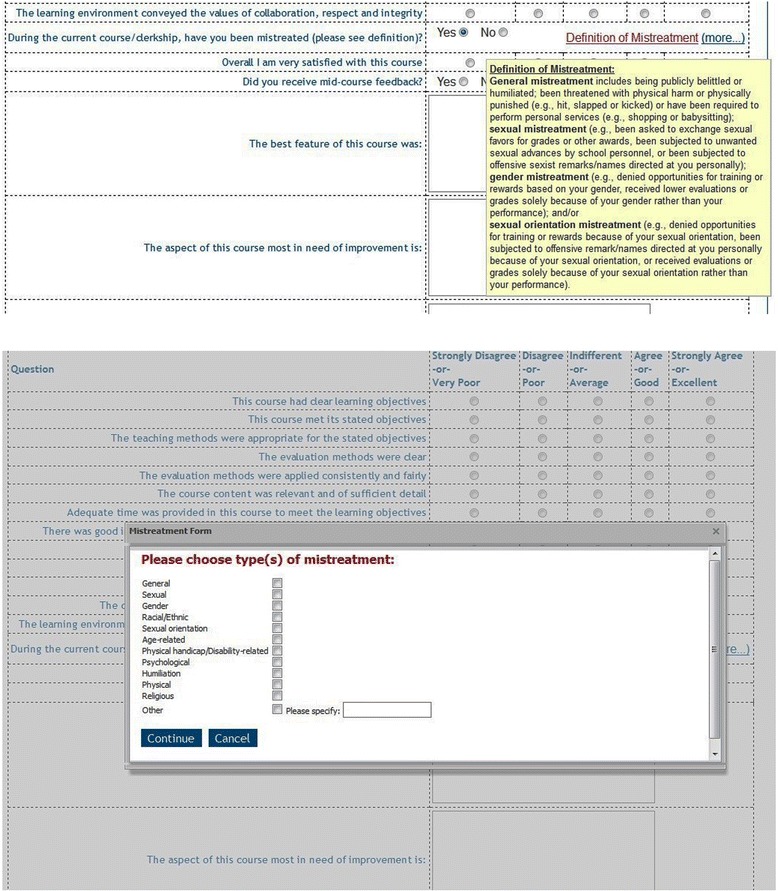
Course/clerkship evaluation form. All students complete a uniform end of course evaluation form (A, top) asking about various components of the course or clerkship. Included in this evaluation are questions about the learning environment and whether s/he has been mistreated. If a student indicates that s/he has been mistreated, a second screen (B, bottom) opens and asks about the type of mistreatment

An essential element for creating a culture of respect, dignity and patient safety is the need for identifiable and anonymous reporting systems [25]. Since fear of reprisal is highly prevalent among trainees who experience mistreatment, we created multiple avenues, both direct face-to-face and online, for students to report mistreatment in confidence or anonymously (Table 1 and ﻿Additional file 1). Confidential face-to-face reports of mistreatment may be made to the Associate Dean for Student Affairs, licensed counselors in Counseling and Psychological Services, clerkship directors, or in periodic student focus groups and clerkship exit interviews with students. The three online avenues for reporting incidents of mistreatment include: 1) a **Professionalism Note** that allows any student, staff member, trainee, or faculty from all health sciences schools to anonymously report unprofessional behavior through a form on the School of Medicine’s homepage; 2) a **Mistreatment Incident Reporting Form** in the student view of our course management system allowing students to report confidentially or anonymously any mistreatment they have experienced or witnessed (Fig. 1); and 3) a newly created section within the mandatory **Course**/**Clerkship Evaluation Form** that asks students about mistreatment and whether or not the learning environment conveyed the institutional values of collaboration, respect, and integrity (Fig. 2a and b). The data collected from these evaluation forms allow aggregate assessment of the prevalence of learning environment concerns.

**Table 1:** Pathways for reporting mistreatment

Pathway	Confidential/anonymous/self-Identified (by student’s choice)	Periodicity/review
Anonymous comments to the Vice Dean Online	Confidential: Anonymous or self-identified by student choice	Report Anytime/Immediate Review
Direct face-to-face	Confidential	Report Anytime/Immediate Review
On-line Professionalism Note	Confidential: Anonymous or self-identified by student choice	Report Anytime/Immediate Review
On-line Mistreatment Incident reporting form	Confidential: Anonymous or self-identified by student choice	Report Anytime/Immediate Review
End of course evaluation	Confidential and anonymous by default	Completed at the end of the course/Reviewed Every 6 Months
Student focus groups and exit interviews; Survey data; AAMC GQ	Confidential, anonymous	Annually/Reviewed within One Month

### Mechanisms for review and adjudication

Another essential element for creating a culture of respect, dignity and patient safety is the need for impartial investigators [25]. Regardless of the mechanism by which an incident is reported, the Associate Dean for Student Affairs serves as the focal point for the initial review of all mistreatment reports. Issues related to physical assault or sexual harassment, workplace violence, Title IX violations, or discrimination are referred immediately to the Office of Institution Diversity and Equity and/or University Police, as appropriate. With regard to issues that are within the Associate Dean’s purview, the Associate Dean attempts to explore and clarify the concern. If the Associate Dean for Student Affairs is unable to resolve the issue between the concerned parties, or if the matter is of a more significant nature, the matter is referred to the Committee on Student Affairs (COSA) for review and adjudication. COSA is a standing committee of the Faculty Senate with multidisciplinary representation from the School of Medicine faculty and students. COSA members are instructed to maintain utmost confidentiality regarding the individuals and issues discussed at their meetings. COSA conducts its formal proceedings to decide the recommended course of action in all such referrals. There is a face-to-face fact-finding meeting of COSA with the student and, subsequently, with the reported individual (faculty member, resident, student, allied health professional, or staff member). At such meetings, additional invitees may include nursing leadership, residents, other students or hospital leadership as appropriate to the case. After review of the facts and deliberations by COSA, the committee makes a recommendation in writing to the Dean of the School of Medicine with copies to other appropriate supervisors and the parties involved. The recommendations of COSA might range from dismissal of the concern, to remediation through educational interventions, counseling and psychological services, to referral to the Office of Institution Diversity and Equity, or referral to the University Hospital Medical Board, referral to Labor Relations for potential suspension or dismissal, referral to University Legal Counsel, referral to University Police, referral to University Community Standards, or other actions as deemed appropriate.

### Monitoring and enforcement

**Fig. 3:**
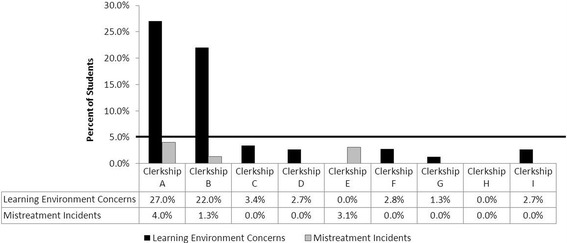
Learning Environment Dashboard: A sample learning environment dashboard report presented to clerkship directors on a semi-annual basis. This dashboard shows the percent of students reporting learning environment concerns and mistreatment incidents across clinical clerkships over a 6-month period. Five percent of students reporting mistreatment incidents was established as the benchmark by stakeholder consensus

The responsibility for enforcement of School of Medicine and University policies relevant to mistreatment in the learning environment and the recommended adjudication of incidents of learner mistreatment sits with the Dean of the School of Medicine and the University official to whom the COSA recommendations are made. Enforcement of these recommendations allows trainees to develop confidence in the process. The avenues for reporting incidents of mistreatment allow us to track patterns and frequency of mistreatment in order to target specific prevention initiatives. Data collected in our course management system help us to identify courses and clerkships in which mistreatment is most frequently reported; this allows targeted educational interventions in those areas. Prior to including specific questions within the end of clerkship mandatory evaluations, we did not have a proactive mechanism to determine exactly where incidents of mistreatment occur on an ongoing basis. Figure 3 is an example of an internal dashboard report using LCME metrics on student learning environment concerns and mistreatment incidents that we provide to clerkship directors on a semi-annual basis. Interestingly, a recent report describing Stanford School of Medicine’s mistreatment prevention program also revealed specific clerkships that deserved diligent educational efforts to improve the learning environment [31]. In addition to our internal data, we also review national comparative data reported through the AAMC GQ, and discuss these metrics publicly in relevant School of Medicine committees.

### Communication and closing the loop

COSA provides periodic reports to the Dean of the Medical School and Faculty Senate on its activities and outcomes. Aggregate reports are made available to School of Medicine educational committees and to the student body. In annual meetings to discuss the Medical Education Summative Evaluation, the Vice Dean for Academic and Faculty Affairs provides specific individualized data to each department chair and course director about mistreatment and learning environment concerns and discusses corrective plans as needed. Monitoring of the learning environment and its positive enhancement is required of all medical schools by the LCME. Each year we report the data from the AAMC GQ as well as our internal end of year three and end of year four survey results to the School of Medicine leadership, various committees within the school, our LCME compliance monitoring leadership team, course and clerkship directors, and the student body.

### Data analyses

Descriptive statistics (percentages) were used to summarize the student response data. Additionally, chi-square tests of significance were performed to compare proportions. A given *p*-value < .05 was considered statistically significant.

## Results

Since the inception of the *WE SMILE* program, our AAMC GQ data show consistent positive trends in both student awareness and reporting of mistreatment incidents. Student awareness of the relevant policies and procedures increased to almost 100% from our earlier data at 67%. Our internal end of year student survey results are also consistent with the GQ data (Table 2, and Additional files 2, 3 and 4).

**Table 2:** AAMC GQ and internal annual end of year survey results on student awareness of mistreatment policies experienced during medical school

Class of	2010	2011	2012	2013	2014	2015	2016
Total # of Students	115	118	123	128	128	121	125
Annual end of preclerkship years survey							
Total # of survey responses	--	--	--	--	--	93	125
Aware of school’s mistreatment policy: “yes”	--	--	--	--	--	92%	98%
Annual end of year three survey							
Total # of survey responses	--	--	--	--	128	114	98
Aware of school’s mistreatment policy: “yes”	--	--	--	--	100%	100%	99%
Annual end of year four survey							
Total # of survey responses	--	--	--	--	95	94	TBD
Aware of school’s mistreatment policy: “yes”	--	--	--	--	99%	100%	TBD
AAMC GQ							
Total # of survey responses	75	84	98	120	111	114	--
Aware of school’s mistreatment policy	51%	71%	98%	100%	100%	100%	--

**Fig. 4:**
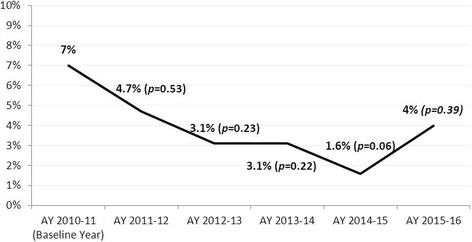
Percent of students reporting mistreatment incidents in Clerkship A over 6 years. Clerkship A had the highest percent of reported mistreatment incidents in AY 2010–11. All other clerkships have had minimal variations over the same time period below the established benchmark of 5%. Chi-square tests of significance were performed to compare proportions. A given *p*-value < .05 was considered statistically significant

There are some reassuring trends in the percentage of reported mistreatment incidents amongst our students. As an example, Clerkship A, with the highest reported mistreatment incidents among all clerkships in AY 2010–11, has shown a consistent downward trend for the past several years (Fig. 4). Using AY 2010–11 as the baseline year, chi-square tests were performed to assess the difference between the percent of students (e.g. 7%) reporting mistreatment incidents at baseline with the student reported percentages in subsequent years. While the differences were not statistically significant, for the most part the trend has been downward with the most recent data (AY 2015–16) showing a slight uptick in such incidents, which serves as a reminder for us to not only proactively monitor such reports but to also continue targeted educational interventions periodically should such upticks occur.

**Fig. 5:**
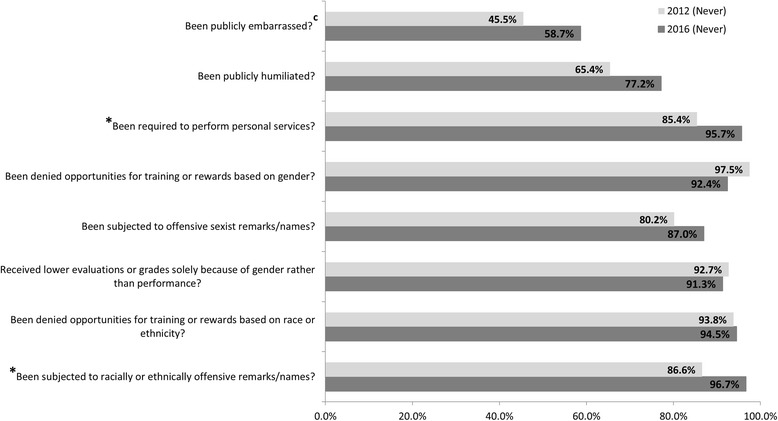
Percent of Students Reporting Never Personally Experiencing Inappropriate Behaviors During Medical School (2012 vs 2016). Behaviors for which over 95% of the respondents (both in 2012 and 2016) reported not having personally experienced are not included in the figure. ^a^ *n* = 81,^b^ *n* = 92, ^c^ Newly added specified negative or offensive behavior in the 2013 AAMC GQ (*n* = 112). * *p* < 0.05, chi-square test

Figure 5 shows the percentage of medical students in our school reporting never having experienced specific behaviors identified by the AAMC GQ (2012: *n* = 81; 2013: *n* = 112; 2016: *n* = 92). It is important to note that in almost all these areas, the percent of students reporting never having experienced such mistreatment behaviors has steadily increased since 2012. Chi-square tests revealed significant differences in student reported areas of personal services performed (*p* = 0.02) and being subjected to racially or ethnically offensive remarks/names (*p* = 0.01). Although not statistically significant, public humiliation and public embarrassment, which are the most commonly reported mistreatment behaviors, have also declined considerably. In 2012, 65.4% of our students reported never experiencing public humiliation whereas in 2016, 77.2% of our students reported so, indicating a 12% improvement over a 4 year span (*p* = 0.09). Similarly, when public embarrassment was added to the AAMC GQ in 2013, only 45.5% of our students reported never having experienced such negative behavior during their time in medical school. In 2016, we saw a 13% increase to 58.7% of students reporting never having experienced public embarrassment as per the AAMC GQ (*p* = 0.09).

In addition, feedback collected from a recent survey of the clinical course directors (13 out of 24, response rate: 54%) indicated that regardless of the mechanism by which an incident is reported, all respondents either ‘strongly agreed’ or ‘somewhat agreed’ that there are formal processes and procedures whereby these incidents are reviewed and adjudicated by the school. All but one respondent either ‘strongly agreed’ or ‘somewhat agreed’ that s/he knew what the mechanisms were for safe reporting of student mistreatment issues and concerns. In terms of our dashboard and annual aggregate reports, a majority of the clinical course directors perceived these as useful in presenting information about student learning environment concerns and student mistreatment data. Interestingly, however, qualitative data gathered from student focus groups and exit interviews were considered as “essential” components of these reports compared to quantitative data collected through surveys such as the end of course evaluations and the AAMC GQ. One respondent commented, “… the feedback is ‘too anonymous.’ “So we get a percent of students who feel the learning environment was not good or there was mistreatment, but there is no way to improve things because there is no detail about what the problem was.” This comment illuminates the importance of narrative data to help expand on quantitative data collected. We have made several positive changes based on the information received from these reports. For example, the data allowed us to initiate frank conversations about student perceptions of supervisor comments, actions and body language. As a result of such conversations, one clerkship developed a mentorship program assigning each student to a resident and faculty mentor to ensure that everyone feels welcomed and included.

## Discussion

### Leadership and its clear message

**Fig. 6:**
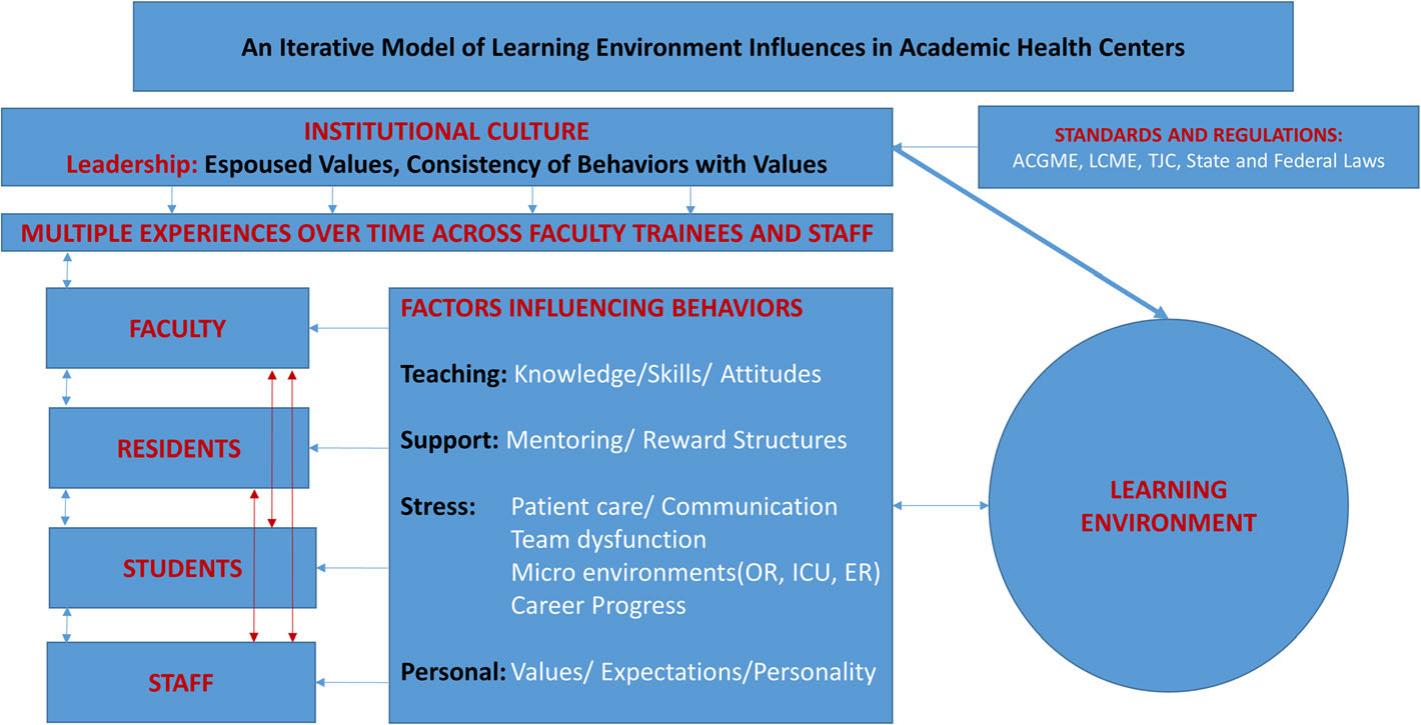
An iterative model of learning environment influences in academic health centers. This model takes into account the external and internal factors involving all stakeholders at different levels of the organization and the iterative nature of these multidirectional influences

The literature on the clinical learning environment has offered many variables that contribute to an unsafe and disrespectful climate: the power differential of the hierarchy; multiple demands on faculty and trainee time and resources; dysfunctional clinical team dynamics; high stress clinical environments; and perceived lack of career and personal support. Institutional leaders have an obligation to establish policies and standards and to communicate these clearly to all stakeholders as critical in maintaining a safe learning environment. As importantly, leaders who espouse the values of respectful behaviors must act accordingly themselves. The behaviors of leaders have a profound influence on the behaviors and attitudes of all other stakeholders, thus impacting the overall institutional culture. Leaders who tolerate inappropriate behaviors send ambiguous messages to faculty regarding behavioral expectations and affect the overall morale by subverting efforts to improve the learning environment. In contrast, leaders whose actions are consistent with the espoused values of the institution and do not tolerate unprofessional behavior set clear expectations with accountability for respectful workplace interactions. Within the mission, vision and values of both quality education and safe clinical care, leadership behaviors must also align with the expectations of external accrediting bodies. We propose a flow model depicting the various factors that converge to influence the learning environment (Fig. 6). At the top of this model is the institutional culture, primarily driven by leadership and influenced by external regulatory agencies, serving as a potent influence on the individuals and their learning/work environments.

In our model, the individuals include faculty, residents, students, and the diverse clinical staff members. A variety of common factors influence the behaviors of all these individuals: teaching or time demands; needs for support and validation; stressors in clinical care; academic or career progress; and personal values. The behaviors of any individual or class of individuals may have a significant influence on the behaviors of all others within the clinical care team, as well as the behaviors of the team as a whole. Learning environments with diminished psychological safety to raise concerns regarding clinical care can result in inadequate communication across care teams leading to adverse patient outcomes. As an example, a faculty member who publicly humiliates a trainee has the potential to impact the behaviors of all trainees and clinical staff present. This, in turn, informs subsequent trainee or staff conduct towards that individual or others. Such iterative influences in clinical team behaviors would be consistent with the argument put forward by Leape et al. that toxic learning environments result in poor communication and unsafe patient care [12]. Trainees accumulate a variety of positive and negative experiences, some more influential than others, that contribute to their professionalism and professional identity formation. Similarly, faculty behaviors may be shaped through experiences with institutional values, trainees, patients, reward systems, and stressors. We see such influences as fitting within a bidirectional, cascading, or transactional framework.

### Lessons learned

#### The gap between anonymous reporting and closing the loop

While we are encouraged by the trends of both AAMC GQ and our own internal data (end of course evaluations, student focus groups, exit interviews), we have found that balancing the need to preserve student anonymity with “closing the loop,” so that students are aware of institutional response and accountability, remains a significant challenge. This need appears to be extremely important for gaining student confidence in the leadership’s commitment to effectively follow-up on reports and to make a meaningful positive impact in the learning environment. However, anonymous student comments do not offer us an opportunity to confidentially close the loop back to the reporter.

#### The value of multiple types and sources of data

By implementing multiple pathways to report mistreatment, we have gained valuable information that would not have been revealed in numeric data from rating scales alone. Although the collection of narrative information is time and labor intensive, it has proven invaluable in enhancing our understanding of student perceptions. For example, we learned that administrators need to be sensitive to student fear of reprisal following reporting of mistreatment. Our experience is consistent with that of Stanford School of Medicine; fear of reprisal is a major concern among students [31]. Our students who worried that their report of mistreatment would have a negative impact on their clerkship grade chose to submit reports through the mistreatment incident reporting form or during end of year focus groups rather than on the end of clerkship evaluation form. Multiple reporting routes provided us converging data that enhanced our awareness of and ability to address specific learning environment concerns. Cumulative summaries provided to all stakeholders on a regular basis permitted the multiple sources and timing of reports to be synthesized effectively for ongoing educational efforts.

#### The utility of a common platform across professions

The availability of the Professionalism Note to any participant in the clinical setting allowed for all members to submit concerns. We believe this is important, as the clinical learning environment concerns often involve negative interprofessional interactions. Opportunities to discuss issues amongst professions through a common forum allows all members to experience a flattened hierarchy and a trustworthy process. Such a process has the potential to address team concerns preemptively to avoid communication challenges that threaten safe patient care.

#### The need for a dynamic process

Programs to improve the learning environment must also be aware of the dynamic nature of teams in the work place. Attention to the annual influx of new trainees and changes in faculty and staff call for timely and periodic education of all stakeholders. Demonstration of a clear commitment to monitor and enhance the learning environment by educational administration communicates its significance to all. Regular discussion of these topics at student meetings is essential to develop student confidence in the system.

## Conclusions

The learning environment within a medical school impacts students’ values, actions, concepts of professionalism, and development of professional identities. Monitoring of the learning environment as required by the LCME is essential to its improvement. Our *WE SMILE* program has demonstrated early success and provides us with cautious optimism for improving our institution’s learning environment. The program is designed to monitor the learning environment, reduce student mistreatment, and be applicable to all undergraduate medical educational experiences. Implementation of this program has afforded us the ability to establish explicit expectations of all educational stakeholders, monitor the learning environment, identify the frequency and sources of mistreatment, and infuse the learning environment with accountability. These early findings in our report, coupled with those of Stanford School of Medicine where a similar approach has been taken, are encouraging in the effort to provide all medical students healthful and exemplary training and practice experiences.

We offer this program as a model for other institutions to consider as they address the challenges of eliminating student mistreatment. It is imperative for educational leaders to recognize that a toxic learning environment perpetuates itself through generations, demoralizes the workforce, affects the educational experiences of trainees and ultimately impacts the safety and quality of patient care.

## Additional files

Additional file 1: Common course evaluation form used at the end of each course and clerkship. (DOCX 16 kb)

Additional file 2: Survey given to students at the end of year 4 of medical school. (DOCX 15 kb)

Additional file 3: Survey given to students at the end of their primary clerkship year (year 3 of medical school). (DOCX 14 kb)

Additional file 4: Survey given to students at the end of year 2 of medical school. (DOCX 14 kb)

## Abbreviations

AAMC: Association of American medical colleges
COSA: Committee on student affairs
GQ: Graduation questionnaire
LCME: Liaison committee on medical education
