# Supplementary material for: A model of influences on the clinical learning environment: the case for change at one U.S. medical school
Source: BMC Med Educ. 2017 Mar 23;17:63. doi: 10.1186/s12909-017-0900-9 (PMC5364543; doi:10.1186/s12909-017-0900-9)
Supplement: Supplementary file 1 — Common course evaluation form used at the end of each course and clerkship. (DOCX 16 kb) [file 12909_2017_900_MOESM1_ESM.docx]

**End of Course Evaluation**

| Question | Strongly Disagree | Disagree | Indiff. | | Agree | | Strongly Agree |
| --- | --- | --- | --- | --- | --- | --- | --- |
| This course had clear learning objectives |  |  |  | |  | |  |
| This course met its stated objectives |  |  |  | |  | |  |
| The teaching methods were appropriate for the stated objectives |  |  |  | |  | |  |
| The evaluation methods were clear |  |  |  | |  | |  |
| The evaluation methods were applied consistently and fairly |  |  |  | |  | |  |
| The course content was relevant and of sufficient detail |  |  |  | |  | |  |
| Adequate time was provided in this course to meet the learning objectives |  |  |  | |  | |  |
| There was good integration of basic science and clinical correlates in this course |  |  |  | |  | |  |
| The learning materials in this course were appropriate |  |  |  | |  | |  |
| Overall, this course was well structured |  |  |  | |  | |  |
| I received timely feedback on my performance in this course |  |  |  | |  | |  |
| The course director was responsive to students' concerns and needs |  |  |  | |  | |  |
| The learning environment conveyed the values of collaboration, respect and integrity |  |  |  | |  | |  |
| Overall I am very satisfied with this course |  |  |  | |  | |  |
| Did you receive mid-course feedback? | | | | Yes | | No | |
| During the current course/clerkship, have you been required to work beyond the SOM stated duty hour limitations? | | | | Yes | | No | |
| Did you report the workload violation to a designated faculty member or a member of medical school administration empowered to handle such complaints? | | | | Yes | | No | |
| During the current course/clerkship, have you been mistreated (please see definition)? | | | | Yes | | No | |
