# Supplementary material for: A model of influences on the clinical learning environment: the case for change at one U.S. medical school
Source: BMC Med Educ. 2017 Mar 23;17:63. doi: 10.1186/s12909-017-0900-9 (PMC5364543; doi:10.1186/s12909-017-0900-9)
Supplement: Supplementary file 3 — Survey given to students at the end of their primary clerkship year (year 3 of medical school). (DOCX 14 kb) [file 12909_2017_900_MOESM3_ESM.docx]

**Annual End of Year Survey on Student Mistreatment and Awareness of Mistreatment Policies**

The school is trying to understand how to improve our educational program for future students. Your feedback will guide us in making slow but steady changes in the right direction. Please be reassured that your feedback is totally confidential and the results are collated before review. The results will be used to implement changes as needed. Thank you for your honest feedback to the school.

--------------------------------------------------------------------------------------------------------------------------

Q. Are you aware that our school has policies regarding the mistreatment of medical students? [Of note, the school has defined mistreatment as behavior that shows disrespect for medical students and unreasonably interferes with their respective learning process. Such behavior may be verbal (swearing, humiliation), emotional (neglect, a hostile environment), and physical (threats, physical harm).]

Yes

No

Q. Have you personally been mistreated during medical school?

Yes

No

Q. Please identify the mistreatment source(s): (check all response categories applicable)

Clinical Faculty (in classroom)

Clinical Faculty (in hospital/clinics)

Residents/Interns

Nurses

Other hospital staff

Administrators

Students

Patients
